# A Case of Plasmablastic Multiple Myeloma With Extramedullary Disease Manifesting as a Myelomatous Pleural Effusion

**DOI:** 10.7759/cureus.32600

**Published:** 2022-12-16

**Authors:** Mohamed Zakee Mohamed Jiffry, Mohammad Ahmed-khan, Napat Rangsipat, Lauren Galligani, Carolina De La Torre

**Affiliations:** 1 Internal Medicine, Danbury Hospital, Danbury, USA; 2 School of Medicine, American University of the Caribbean, Cupecoy, SXM

**Keywords:** malignant hematology, relapsed refractory multiple myeloma, myelomatous pleural effusion, extra-medullary myeloma, multiple myeloma

## Abstract

Multiple myeloma (MM) is a neoplasm of plasma cell origin characterized by the proliferation of immunoglobulin-producing plasma cells in the bone marrow. Extramedullary disease (EMD) occurs in approximately 10% of patients with MM. Myelomatous pleural effusion (MPE) is a possible manifestation of EMD and has been associated with a poorer prognosis.

A 66-year-old female was evaluated after an abnormal serum protein electrophoresis that showed a 2.1 g/dL M-spike in the gamma region, highly suggestive of plasma cell dyscrasia. Imaging subsequently confirmed the bony metastasis. A bone marrow biopsy confirmed plasmablastic MM, and she was started on chemotherapy. She presented three months later with bilateral pleural effusions, with cytology revealing neoplastic plasmacytoid cells. Despite transitioning to dexamethasone, cyclophosphamide, etoposide, and cisplatin (the V-DCEP regimen) due to disease progression, her myeloma remained refractory to treatment, and she expired one month later.

MPE in MM is associated with a poor prognosis, with a median overall survival (OS) of 13 months in MPE compared to 37 months in other EMDs. A higher tumor burden and greater multisite extra-medullary lesions are also characteristics of MPE in MM. There is no standard of care for the management of EMD, and salvage regimens such as RVD and V-DCEP are commonly employed. The management of MM with EMD remains a challenge, and more investigation is required before effective treatment regimens may be employed in this setting.

## Introduction

Multiple myeloma (MM) is a neoplasm of plasma cell origin characterized by the proliferation of immunoglobulin-producing plasma cells in the bone marrow. A diagnosis of MM is made when a bone marrow biopsy demonstrates at least 10% clonality of plasma cells and one or more myeloma-defining events, such as end-organ damage attributed to the proliferative disorder. Extramedullary disease (EMD) occurs in approximately 10% of patients with MM and is defined as an aggressive soft tissue plasmacytoma of malignant clonal cells that grow independently of the initial bone marrow neoplasm [1-3]. Extramedullary involvement often occurs via hematogenous spread into distant organs or soft tissues, including the liver and pleura, and is more prevalent in refractory than newly diagnosed MM [4]. Myelomatous pleural effusion (MPE) is a possible manifestation of EMD. Although MPE only occurs in <1% of patients with MM, it has been associated with a poorer prognosis than MM alone or combined with EMD in other tissues and a marked decrease in survival from the onset of pleural involvement [5]. It is therefore clinically important to identify and treat pleural effusions caused by EMD in a timely manner. We present the case of a 66-year-old female who presented with MPE after multiple treatment regimens for refractory MM.

## Case presentation

A 66-year-old female was evaluated by hematology/oncology for an abnormal serum protein electrophoresis. She was noted to have a 2.1 g/dL M-spike in the gamma region, highly suggestive of a plasma cell dyscrasia. A serum immunofixation test showed IgG-type lambda monoclonal gammopathy. At the time, the patient was able to perform her activities of daily living without hindrance with an ECOG of 0 (fully active with no performance restrictions) and denied symptoms including unexplained pains, fevers, chills, loss of weight, or loss of appetite.

**Figure 1:**
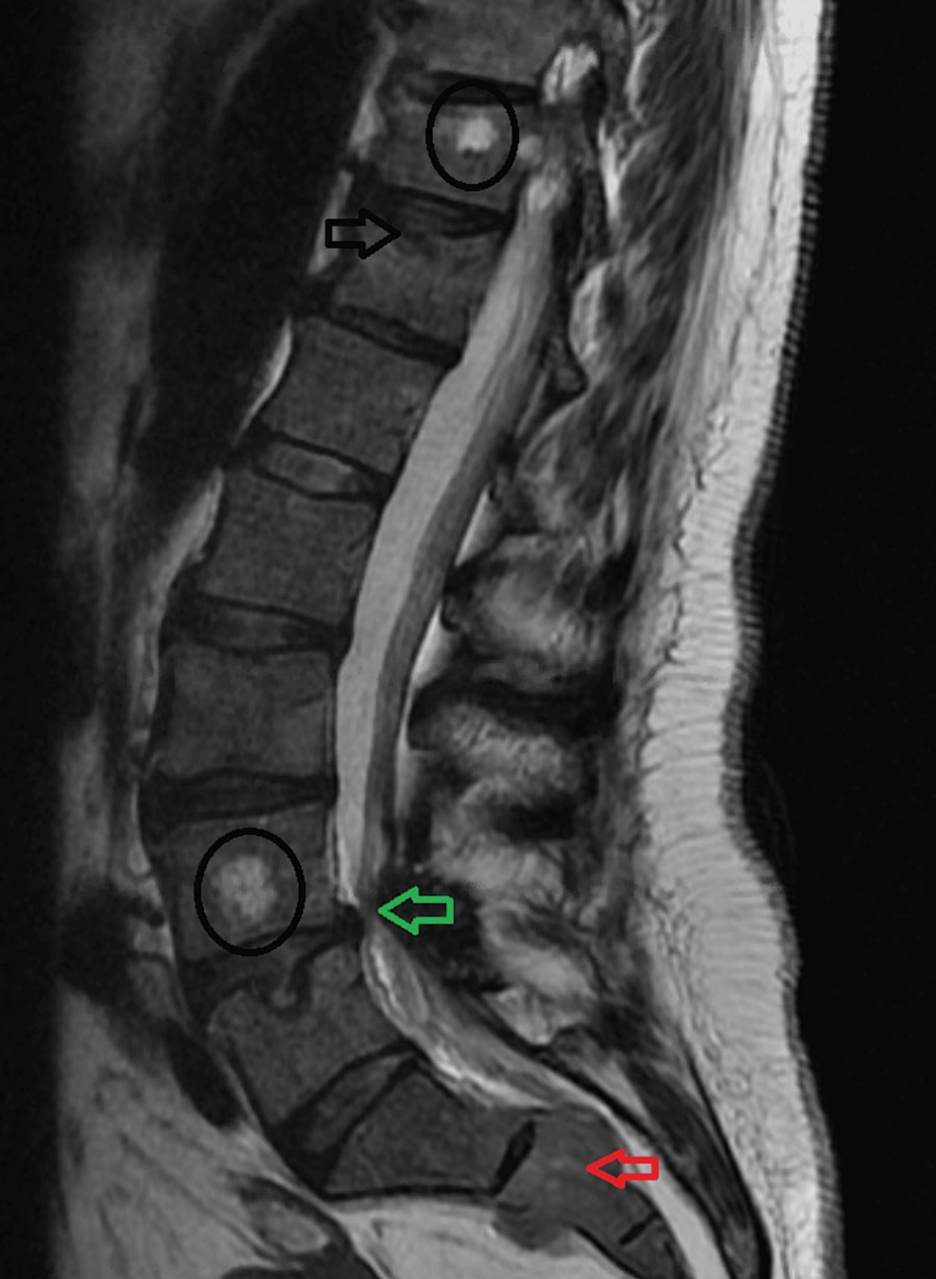
T2-weighted sagittal magnetic resonance imaging of the lumbar spine. *Black circles*: Multiple metastatic lesions identified in the thoracic and lumbar vertebral column. *Black arrow:* Wedge compression fracture of T12 vertebral body. *Green arrow:* Central canal stenosis at the L4-L5 intervertebral space. *Red arrow:* Large lytic lesion identified at the S2 level.

**Figure 2:**
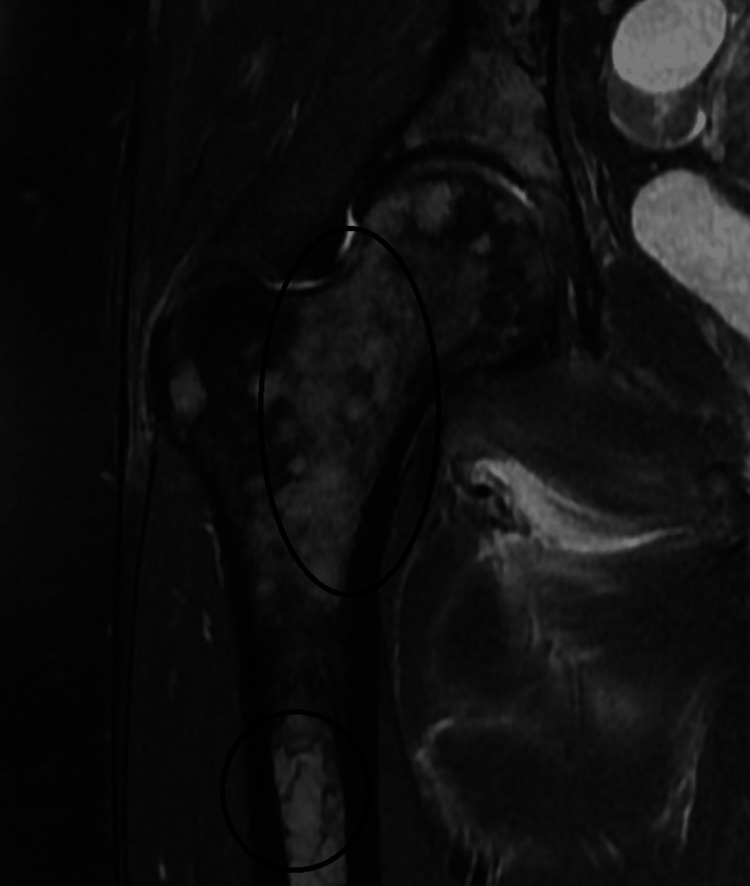
T2-weighted coronal magnetic resonance imaging of the right proximal femur. *Black circles*: Extensive marrow replacement process visualized in the proximal femur.

The patient was subsequently lost to follow-up. Eight months later, she presented to the emergency room complaining of sciatica in her right leg. Imaging showed extensive metastatic disease involving the spine with multiple large lytic lesions (Figure 1). Imaging also showed pathologic marrow replacement from MM (Figure 2).

A complete blood count (CBC) showed normocytic anemia with a hemoglobin of 9.7 g/dL. A comprehensive metabolic panel (CMP) was unremarkable for hypercalcemia or derangement of renal functions. Repeat SPEP identified a 1.8 g/dL M-spike in the gamma region.

A bone marrow aspirate and biopsy were performed, which showed greater than 90% neoplastic plasma cells. Flow cytometric analysis confirmed lambda-light chain-restricted monoclonal plasma cells. Cytogenetic analysis revealed multiple chromosomal abnormalities, including a deletion of the short arm of chromosome 17, a gain of function in chromosome 1q, and a monosomy 13.

She was subsequently initiated on an RVD regimen with bortezomib, lenalidomide, and dexamethasone, with regular follow-ups being scheduled. She appeared to tolerate chemotherapy and successfully completed 10 cycles of treatment over the course of the year without significant toxicity or worsening symptoms. A repeat bone marrow biopsy after the 10th cycle showed worsening marrow involvement of approximately 95% plasma cells, precluding a bone marrow transplant at the time. During her 11th cycle, she developed worsening right lower limb sciatica. A repeat MRI of the lumbar spine showed substantial interval worsening and tumor involvement of the sacrum, with extensive tumor filling of the sacral spinal canal obliterating the thecal sac and extending to the right L5-S1.

The patient was referred to radiation oncology for radiotherapy (RT) of the ischium and L5 sacrum, which she successfully completed with significant pain relief. A repeat SPEP at this time showed a worsening M-spike of 2.4 g/dL in the gamma region. The decision to add daratumumab was made, and bortezomib was reduced to once-weekly dosing. Despite one cycle of the adjusted regimen, she continued to deteriorate clinically with worsening fatigue, intermittent fevers, symptomatic hypercalcemia, and recurrent hospitalizations for peritonsillar abscesses requiring multiple antibiotic courses, as well as a DVT of her right lower extremity for which she was initiated on rivaroxaban.

**Figure 3:**
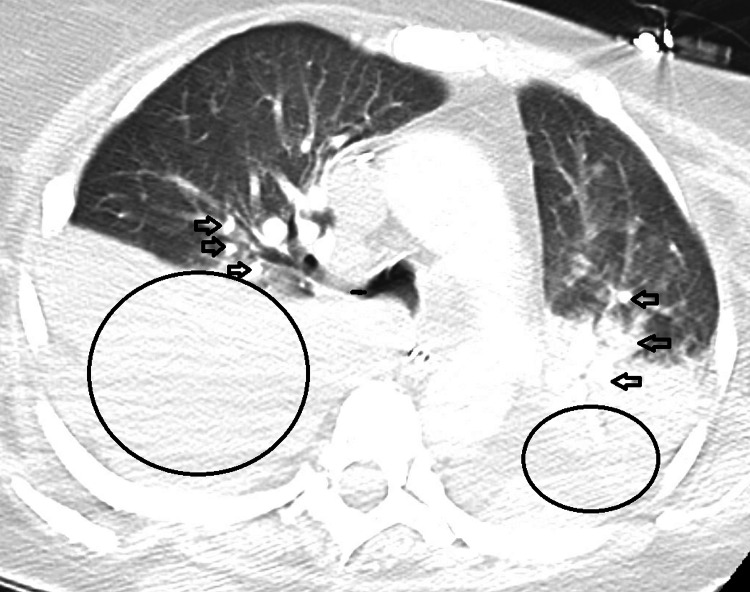
Computed tomography scan of the chest. Black circles: Bilateral pleural effusions were identified, right side significantly greater than the left. Black arrows: Areas of nodular hyperdensities are recognized bilaterally with lobular infiltration evident as well.

A CT chest was performed after she presented with the complaint of shortness of breath for a one-week duration with worsening fatigue, which revealed bilateral pleural effusions and new areas of lobular and nodular pleural densities consistent with pleural metastasis (Figure 3). CT scan of the liver also demonstrated multiple hepatic masses.

She underwent a therapeutic thoracentesis with the evacuation of 850 cc of amber fluid. Pleural fluid analysis with immunohistochemical staining analysis showed neoplastic plasmacytoid cells positive for CD138 and CD56, which supported a diagnosis of malignant pleural effusion from a known plasma cell neoplasm. The pleural fluid tested negative for Epstein-Barr encoded RNA in situ hybridization and HHV-8 immunostaining. The pleural fluid analysis was consistent with Lights' criteria for exudative-type effusion (Table 1).

**Table 1:** Pleural fluid analysis with serum comparators demonstrating two out of three Light's criteria being positive, i.e., effusion protein/serum protein ratio greater than 0.5 and effusion LDH level greater than two-thirds the upper limit of the laboratory's reference range of serum LDH. LDH: lactate dehydrogenase.

Laboratory value	Pleural fluid	Serum (reference range)
Total nucleated cells (/cumm)	1335	-
White blood cells (/cumm)	222	-
Polymorphonuclear leukocytes (%)	10	-
Monocytes (%)	90	-
Red blood cells (/cumm)	5,000	-
Glucose (mg/dL)	160	129 (70–99)
Lactate dehydrogenase (U/L)	497	1,000 (122–222)
Total protein (g/dL)	5.3	7.3 (6.3–7.9)

A liver biopsy of the hepatic masses showed atypical plasma cell proliferation most consistent with plasmablastic myeloma. A repeat bone marrow biopsy once more showed hypercellular bone marrow with approximately 95% involvement by the known plasma cell dyscrasia, with flow cytometry positive for the IgG lambda plasma cell population. In view of her progressive and refractory myeloma, she received one cycle of the V-DCEP regimen (bortezomib with dexamethasone, cyclophosphamide, etoposide, and cisplatin).

The repeat bone marrow biopsy demonstrated greater than 90% involvement by MM, once again precluding bone marrow transplantation. The patient experienced worsening fatigue, a poor appetite, and marked weight loss. She was readmitted a month later for evaluation of profound weakness, dehydration with poor oral intake, and cachexia. Her goals of care were transitioned to comfort measures only, and the patient subsequently expired following respiratory failure.

## Discussion

Myelomatous pleural effusion accounts for less than 1% of pleural effusion in MM. A prospective cohort study over a 10-year period discussed the incidence of MPE among 3,480 patients with pleural effusion and 319 patients with multiple myeloma and found that only two cases, or 0.6% of MM cases, had MPE [6].

The majority of pleural effusions found in multiple myeloma are non-myelomatous [7]. Pleural effusion of various causes can be found in up to 13% of cases, including parapneumonic, reactive, renal, CHF, TB, and MPE, with MPE rendering a poorer outcome in comparison. A study found median overall survival in MPE was 13.0 months, compared to 37.0 months in other EMD and 60.6 months in MM without EMD, with MPE patients being found to have a higher level of beta 2-microglobulin tumor burden and greater multisite extramedullary lesions [5].

Hepatic involvement in EMD is a rare occurrence and indicates a poor prognosis, although large-scale studies are limited due to the rarity of this phenomenon. A study comparing the incidence and pattern of liver involvement in hematologic malignancies discusses infiltration of the liver in MM as being less common than other hematologic malignancies and notes that sinusoidal involvement with nodular infiltration is the usual pattern when such involvement occurs [8]. A study of GI involvement in MM showed a disproportionately high percentage of MM with plasmablastic morphology compared to the general population of MM (29% vs 2%, respectively), a morphology that our case demonstrated [9].

Cytologic markers considered high risk that were identified in our patient included a deletion of the short arm of chromosome 17, a gain of function in chromosome 1q, and a monosomy 13. Deletion of 17p is seen in 5-10% of patients with MM and is associated with TP53 gene mutations [10,11]. A gain of function in chromosome 1q is a common genetic mutation, found in 40% of MM patients at diagnosis. It has been associated with disease progression and drug resistance [12]. Existing literature is sparse regarding the association between high-risk cytologic markers and the development of EMD.

There is no standard treatment approach for the management of extramedullary myeloma. Induction of chemotherapy with novel agents like thalidomide, lenalidomide, and bortezomib is commonly used. The RVD regimen (lenalidomide, bortezomib, and dexamethasone) is commonly used as induction therapy and as a salvage regimen for relapsed or refractory cases of multiple myeloma, regardless of transplant eligibility. A study of a cohort of 1,000 MM patients treated with the RVD regimen showed a higher response rate and longer survival time, with the median overall survival (OS) time for this cohort being 126.6 months [13].

V-DCEP (bortezomib, cyclophosphamide, etoposide, and cisplatin) is another regimen employed in the salvage setting, with a reported median OS of 49 months when employed as salvage therapy per a retrospective study of relapsed/refractory MM patients [14]. RT has also shown great efficacy when used as a pain control measure in MM. A retrospective review of 449 MM patients who received RT reported a 76.4% complete relief of pain, although a clear survival benefit was not demonstrated [15].

## Conclusions

This case demonstrates the clinical heterogeneity of patients manifesting EMD in MM. Although pleural effusions may occur in MM patients for a variety of reasons, MPE is a distinct entity that portends a poor outcome and needs to be recognized as such. MM with plasmablastic morphology may also be more likely to involve the GI tract, as evidenced by the liver metastasis noted in our case.

Overall, the management of MM patients with EMD remains a challenge with no evidence to guide optimal therapies. Of particular interest, genetically modified T-cells and chimeric antigen receptor T-cell (CAR-T) therapy represent a possible modality for treatment in the future.
